# Supplementary material for: Elevated ANG2/ACE2 and immune responses associated with Plasmodium falciparum and SARS-CoV-2 coinfection in Cameroon
Source: PLOS Glob Public Health. 2026 Feb 27;6(2):e0005010. doi: 10.1371/journal.pgph.0005010 (PMC12948122; doi:10.1371/journal.pgph.0005010)
Supplement: Attachment — Submitted filename: Comments_VS.docx [file pgph.0005010.s002.docx]

Investigating the potential of clinical variables and ace2/tmprss2 genetic variants as a biomarkers for COVId-19 and malaria co-infection Cameroon.

PLOS Global Public Health

PGPH-D-25-01398

The research topic is interesting but it has major concerns. The research work reported here does not fully align with the title of the paper. The data does not clearly grasp the title and discuss the aspects of the title in the manuscript and have been vague about it. There are several grammatical errors and spelling errors too. Aims need to be modified. Discussion needs to be shortened and does not discuss much about the genetic variants. In general the introduction, results and discussion are not in sync with the title, unable to give a clear understanding to the reader about the biomarkers or genetic variants for both the diseases. In light of these comments I do not recommend this paper.

**Other comments:**

1. As per the title of the paper “Investigating the potential of clinical variables and ace2/tmprss2 genetic variants as a biomarkers for COVId-19 and malaria co-infection Cameroon,” does not explain the genetic variables in a clear manner in the introduction nor in the discussion.
2. Short title has error ‘alta’ which needs to be corrected.
3. The scientific names are not italicized eg, Plasmodium and several places Covid-19 is written in different way thought out the manuscript.

**Introduction-**

4. In Introduction the background is vague. No mentioned of any previous SNP based study is there.
5. Which SNPs are to be studied do not find mention in the introduction.
6. Line 91 – ‘Trained immunity’. Please rephrase the sentence.
7. Hypothesis is not clear. Pl revise.
8. Aims need to be modified.

**M & M-**

9. The subheadings are very long. They need to be crisper.
10. Line 126 - - ‘as if 2015’ this is an old data date, to be replaced with current.
11. Full stops to be added after completion of sentences.
12. Line 154 – ‘malaria tests were ordered’ – It is not clear which test were undertaken.
13. Line 157 – ‘aliquoted samples’ – not clear with aliquoted samples.
14. Line 168 ‘RT-PCR for cox-3 gene’ done Any particular reason why this test was used. Explain it.
15. No clear mention of which species was studied here.
16. Title mentions malaria patients so it is expected that Pf and non- Pf species should all be included here, but this does not seem so. Pl explain this clearly.
17. Line 176 no reference mentioned.

**Results -**

18. Table -1 ‘Malaria mono’ specify clearly.

19. Headings are too long, needs to be shortened. Heading itself is stating the results.
20. Line 319 – homo, heterozygous and homozygous mutant not explained properly.

**Discussion –**

21. Too long. Needs to be fully revised.
22. Lines 377-380. Contradicting and derived statement needs to be revisited.
23. Lines 386-387. The link of malaria, AST ALT increased levels with Schistosomiasis is not clearly understandable.
