# Supplementary material for: Elevated ANG2/ACE2 and immune responses associated with Plasmodium falciparum and SARS-CoV-2 coinfection in Cameroon
Source: PLOS Glob Public Health. 2026 Feb 27;6(2):e0005010. doi: 10.1371/journal.pgph.0005010 (PMC12948122; doi:10.1371/journal.pgph.0005010)
Supplement: Attachment — Submitted filename: Comments PGPH.docx [file pgph.0005010.s003.docx]

Comments regarding Reviewer Assignment for PGPH-D-25-01398 - [EMID:78717a945af9af42]

1. English writing is poor, there are grammatical mistake throughout the text. Authors should improve the English writing. This manuscript is required the major revision.
2. The sentence “ Malaria is a tropical disease caused by parasites transmitted through bites from Plasmodium-infected mosquitoes resulting in approximately 249 million cases and 608,000 deaths annually, particularly in sub-Saharan Africa” needs to be reframed. It is not telling about the mosquito species. Please mention WHO report and year regarding malaria global burden and deaths.
3. Please cite , any meta -analysis or original research work on the trained immunity induced by previous malaria exposure, which protects against covid-19 severity.
4. Covid severity is caused by due cytokine storm of pro-inflammatory cytokines. How can pro-inflammatory cytokines induce during malaria protect against covid severity. Line 96-98
5. Authors should use the reference, alternate genotypes in place of wild and mutant. Line 318-19
6. Authors should add the study design and prevalence of malaria and covid-19 in the Cameroon.
