# Supplementary material for: Elevated ANG2/ACE2 and immune responses associated with Plasmodium falciparum and SARS-CoV-2 coinfection in Cameroon
Source: PLOS Glob Public Health. 2026 Feb 27;6(2):e0005010. doi: 10.1371/journal.pgph.0005010 (PMC12948122; doi:10.1371/journal.pgph.0005010)
Supplement: Attachment — Submitted filename: Response to Reviewers.docx [file pgph.0005010.s004.docx]

*Republic of Cameroon*

*University of Yaoundé I*

*Faculty of Medicine and Biomedical Sciences*

*Department of Biochemistry*

*P. O Box 812 Yaoundé*

*Tel: +237 22 23 95 84*

République du Cameroun

Université De Yaoundé I

Faculté de médicine et des Sciences Biomédicales

Département de Biochimie

B. P. 812 Yaoundé, Cameroun

Tel: +237 22 23 95 84

--------------------------------------------------------------------------------------------------------------------------

November 7^th^, 2025

Dear Editorial team,

Thank you for the opportunity to revise our manuscript titled “**Elevated ANG2/ACE2 and Immune Responses Associated with *Plasmodium* and SARS-CoV-2 Co-infection in Cameroon**” (PGPH-D-25-01398). We appreciate the constructive feedback provided by the reviewers, which has been invaluable in improving the quality of our work. Below, we provide our responses to each point raised.

**Responses to Journal Requirements:**

**Journal Requirements:**

**1**. Thank you for bringing this to our attention. This study was funded by the African coaLition for Epidemic Research, Response and Training (ALERRT), part of the EDCTP2 Programme supported by the European Union under grant agreement RIA2016E-1612. ALERRT is also supported by the United Kingdom National Institute for Health Research and the Wellcome Trust (Ref 221012/Z/20/Z). The funders had no role in the study design, data collection and analysis, decision to publish, or preparation of the manuscript.

**2**. Thank you for bringing this to our attention. All data supporting the findings of this study are available within the manuscript and the supporting information files.

**3.** Thank you for this comment. We have carefully reviewed all figures and materials included in our submission. All content used in the manuscript is original and created by the authors, and no copyrighted material requiring permission has been included. Therefore, no additional permissions are necessary under the PLOS copyright policy.

We appreciate the clarification regarding copyright requirements. Figure 1, which contained the map in question, has been removed from the revised version of the manuscript to ensure full compliance with the PLOS copyright policy.

**4.** Thank you for your helpful comment. All trademark and copyright symbols (® and ™) have been carefully reviewed and removed from the manuscript, including those on pages 5 and 6, in accordance with the journal’s guidelines.

**5.** Thank you for the suggestion. We have carefully reviewed the recommended publications and evaluated their relevance to our study. Based on this assessment, we have cited the relevant works where appropriate in the revised manuscript.

**Reviewers’ comments**

**Responses to Reviewer #1:**

1. Thank you for your valuable feedback. Based on your suggestion, we have revised the title to “**Elevated ANG2/ACE2 and Immune Responses Associated with Plasmodium and SARS-CoV-2 Coinfection in Cameroon**”. This new title more clearly reflects the focus of the study. Additionally, we have revised the introduction and discussion sections.
2. Thank you for pointing this out. We have corrected the error in the short title, changing "alta" to “alter” on the PLoS platform
3. Thank you for your observation. We have revised the manuscript to ensure that the scientific names, such as *Plasmodium*, are properly italicized. Additionally, we have standardized the writing of COVID-19 throughout the manuscript. Line 1, 75,76, 90, 97 101, 265 …

**Introduction:**

4. Thank you for your feedback. We have revised the introduction to provide a clearer and more detailed background to better set the context for the study.
5. Thank you for your comment. We have removed the section on SNPs from this manuscript, which is why they are no longer mentioned in the document
6. Thank you for your suggestion. We have rephrased the sentence by replacing “trained immunity” with “long-lasting innate immune protection” to improve clarity. On page 3, line 92
7. Thank you for your valuable comment. We have revised the manuscript to clarify the study hypothesis. The hypothesis is now clearly stated in the Introduction section to better reflect the objectives and rationale of the study. On page 3, line 107-108.
8. Thank you for your comment. We have revised the aim of the study to make it clearer and better aligned with the objectives of the research. Line 109-111

**Methodology:**

9. Thank you for your feedback. We have revised the subheadings to make them more concise and clearer in the entire document.
10. Thank you for your insightful feedback. We acknowledge that the reference "2015" has already been updated with the most current data available. On line 134
11. Thank you for your constructive feedback. We have carefully reviewed the manuscript and added full stops at the end of all completed sentences, as suggested. We appreciate your attention to detail and have made the necessary corrections in the revised manuscript.
12. Thank you for your constructive feedback. In response to your comment, we have clarified the sentence on line 154
13. Thank you for your helpful feedback. In response to your comment, we have revised the manuscript for clarity. The term "aliquoted samples" has been further explained to specify that it refers to the serum and plasma samples. One line 158
14. Thank you for your insightful question. The particular reason for using RT-PCR targeting the **cox-3 gene** in malaria diagnosis is that this gene exists in multiple copies per parasite. This feature enhances both the sensitivity and specificity of the detection, particularly in low-parasitemia samples, where other diagnostic methods may be less effective.
15. We appreciate the reviewer’s observation. We have clarified in the manuscript that *Plasmodium falciparum* is the species investigated in this study.
16. We thank the reviewer for this valuable comment. We would like to clarify that our study focused on *Plasmodium falciparum* species and we have revised the title to indicate that.
17. Thank you for pointing this out. We have added the appropriate reference to support this statement on line 178.

**Results:**

18. Thank you for bringing this to our attention. We have revised the term *“Malaria mono”* in Table 1 to *“Malaria (+)”* for clarity.
19. Thank you for bringing this to our attention. We have revised the section headings throughout the manuscript to make them more concise and descriptive.
20. Thank you for your valuable comment regarding the clarification of homozygous wild type, heterozygous, and homozygous mutant genotypes. We agree that this section required explanation that is more detailed. However, after revising the focus of the manuscript, we have removed the polymorphism-related analysis entirely from the current version.

**Discussion:**

21. Thank you for highlighting the concern about discussion length. We appreciate your feedback. The discussion has now been carefully revised and significantly shortened
22. Thank you for pointing out the issue regarding contradictory and derivative statements. We carefully re-examined the relevant sections and revised the text to ensure scientific consistency and clarity.
23. Thank you for your comment regarding the link between malaria, elevated AST/ALT levels, and schistosomiasis. The discussion has been revised to better clarification.

**Responses to Reviewer #2:**

1. Thank you for your valuable feedback concerning the quality of English language and grammar. The entire manuscript has now undergone thorough language revision, including grammatical corrections, restructuring of unclear sentences, and enhancement of overall flow.
2. Thank you for your observation. We agree that specifying the mosquito genus and updating the global burden data improves precision. The sentence has been revised line 75-77
3. Thank you for your comment. We have already cited a relevant systematic review on trained immunity induced by prior malaria exposure and its potential protective effect against COVID-19 severity (see citation number 10).
4. Thank you for this important comment. Severe COVID-19 is driven by an excessive “cytokine storm,” In malaria, early induction of pro-inflammatory cytokines such as IFN-γ and IL-1β contributes to rapid pathogen recognition and antiviral defenses. This early innate priming may help contain SARS-CoV-2 replication before it escalates into uncontrolled inflammation.
5. Thank you for your valuable comment. We have removed the polymorphism-related analysis entirely from the current version.
6. Thank you for this valuable suggestion. We have added the prevalence and mortality of malaria and covid-19 in Cameroon to provide appropriate epidemiological context. One line 81-83

We believe that these revisions have addressed all the comments and suggestions from the reviewers, and we are grateful for their constructive feedback which has substantially improved the manuscript. Thank you once again for your consideration. We look forward to your positive response.

Sincerely

Palmer Masumbe Netongo

[masumben@gmail.com](mailto:masumben@gmail.com)

[masumbe.palmer@facsciences-uy1.cm](mailto:masumbe.palmer@facsciences-uy1.cm)

(+237) 677613131/ 699868190
