# Supplementary material for: Elevated ANG2/ACE2 and immune responses associated with Plasmodium falciparum and SARS-CoV-2 coinfection in Cameroon
Source: PLOS Glob Public Health. 2026 Feb 27;6(2):e0005010. doi: 10.1371/journal.pgph.0005010 (PMC12948122; doi:10.1371/journal.pgph.0005010)
Supplement: Attachment — Submitted filename: Response_to_Reviewers_auresp_2.docx [file pgph.0005010.s006.docx]

*Republic of Cameroon*

*University of Yaoundé I*

*Faculty of Medicine and Biomedical Sciences*

*Department of Biochemistry*

*P. O Box 812 Yaoundé*

*Tel: +237 22 23 95 84*

République du Cameroun

Université De Yaoundé I

Faculté de médicine et des Sciences Biomédicales

Département de Biochimie

B. P. 812 Yaoundé, Cameroun

Tel: +237 22 23 95 84

--------------------------------------------------------------------------------------------------------------------------

January 8^th^, 2026

Dear Editorial team,

Thank you for the opportunity to revise our manuscript titled “**Elevated ANG2/ACE2 and Immune Responses Associated with *Plasmodium* and SARS-CoV-2 Co-infection in Cameroon**” (PGPH-D-25-01398). We appreciate the constructive feedback provided by the reviewers, which has been invaluable in improving the quality of our work. Below, we provide our responses to each point raised.

**Responses to Journal Requirements:**

**Journal Requirements:**

**1.** Financial Disclosure statement.

a. We are grateful for raising this point. The manuscript has been updated to include the initials of the relevant authors next to each funding source, clearly indicating which authors were supported by each grant.

b. Thank you for this important comment. The funders contributed to the all process including the study design, data collection and analysis, decision to publish, and preparation of the manuscript.

**2.** We are grateful for pointing this out. The funding details have been thoroughly checked and updated so that the funders and grant numbers match exactly between the Financial Disclosure section and the Funding Information tab, with a consistent ordering in both places.

**3.** Thank you for your suggestion. The authors have declared that no competing interests exist.

**4.** Thank you for this comment. We confirm that all raw data required to replicate the results of this study (The means, standard deviations and other measures), constituting the minimal data set as defined by PLOS, are included in the submission as Supporting Information and the Data Availability Statement has been updated accordingly.

**5.** Thank you for bringing this to our attention. The manuscript has been revised to ensure that Figure 1 is explicitly and correctly referenced in the text to facilitate proper linking. Line 138

**6.** Thank you for your helpful comment. The articles suggested by the reviewer have been cited on line 98 for the paper **PMID: 39052480** and line 336 **for** the paper **PMID: 36415655**

**7.** Thank you for bringing this to our attention. We have carefully reviewed and corrected the reference list to ensure that it is complete and accurate. No retracted articles are cited in the revised manuscript. All changes to the reference list have been addressed in this rebuttal letter.

We believe that these revisions have addressed all the comments and suggestions from the reviewers, and we are grateful for their constructive feedback which has substantially improved the manuscript. Thank you once again for your consideration. We look forward to your positive response.

Sincerely

Palmer Masumbe Netongo

[masumben@gmail.com](mailto:masumben@gmail.com)

[masumbe.palmer@facsciences-uy1.cm](mailto:masumbe.palmer@facsciences-uy1.cm)

(+237) 677613131/ 699868190
