# Supplementary material for: Elevated ANG2/ACE2 and immune responses associated with Plasmodium falciparum and SARS-CoV-2 coinfection in Cameroon
Source: PLOS Glob Public Health. 2026 Feb 27;6(2):e0005010. doi: 10.1371/journal.pgph.0005010 (PMC12948122; doi:10.1371/journal.pgph.0005010)
Supplement: Attachment — Submitted filename: Reviewer Comments PGPH revision.docx [file pgph.0005010.s005.docx]

Reviewer Comments

The authors have revised the manuscript as per comments. I would recommend it with a minor revision.

1. Authors may cite the paper **PMID: 39052480** at line no 98-99 regarding the malaria induced cross protection against COVID19, and cite another paper **PMID: 36415655** regarding cytokine upregulation during COVID 19 and malaria diseases.
